# Pressure‐Induced Remarkable Spectral Red‐Shift in Mn^2+^‐Activated NaY_9_(SiO_4_)_6_O_2_ Red‐Emitting Phosphors for High‐Sensitive Optical Manometry

**DOI:** 10.1002/advs.202308221

**Published:** 2023-12-16

**Authors:** Qifeng Zeng, Marcin Runowski, Junpeng Xue, Laihui Luo, Lukasz Marciniak, Víctor Lavín, Peng Du

**Affiliations:** ^1^ School of Physical Science and Technology Ningbo University Ningbo Zhejiang 315211 China; ^2^ Faculty of Chemistry Adam Mickiewicz University Uniwersytetu Poznańskiego 8 Poznań 61–614 Poland; ^3^ School of Science Jiangsu University of Science and Technology Zhenjiang 212100 China; ^4^ Institute of Low Temperature and Structure Research Polish Academy of Sciences Okólna 2 Wrocław 50–422 Poland; ^5^ Departamento de Física MALTA‐Consilider Team Universidad de La Laguna Apartado de Correos 456 San Cristóbal de La Laguna Santa Cruz de Tenerife E‐38200 Spain

**Keywords:** d‐block metal ions, high‐pressure luminescence, Mn^2+^ emission, optical manometry, phosphors

## Abstract

To settle the low sensitivity of luminescent manometers, the Mn^2+^‐activated NaY_9_(SiO_4_)_6_O_2_ red‐emitting phosphors with splendid pressure sensing performances are developed. Excited by 408 nm, the resulting products emit bright red emission originating from ^4^T_1_(^4^G) → ^6^A_1_ transition of Mn^2+^, in which the optimal concentration of the activator ion is ≈1 mol%. Moreover, the admirable thermal stability of the developed phosphors is studied and confirmed by the temperature‐dependent emission spectra, based on which the activation energy is derived to be 0.275 eV. By analyzing the pressure‐dependent Raman spectra, the structural stability of the synthesized compounds at extreme conditions is verified. Furthermore, the designed phosphors exhibit remarkable spectral red‐shift at elevated pressure. Especially, as pressure increases from 0.75 to 7.16 GPa, the emission band centroid shifts from 617.2 to 663.4 nm, resulting in a high sensitivity (d*λ*/d*P*) of 7.00 nm GPa^−1^, whereas the full width at half maximum (FWHM) increases from 83.0 to 110.6 nm, leading to the ultra‐high sensitivity (dFWHM/d*P*) of 10.13 nm GPa^−1^. These achievements manifest that the designed red‐emitting phosphors are appropriate for ultrasensitive optical manometry. More importantly, the developed manometer is a current global leader in sensitivity, when operating in the band‐width mode, that is, FWHM.

## Graphical abstract

The designed luminescent materials do not only show visible red emission upon NUV light excitation, but also are potential candidates for dual‐mode optical manometry, which exhibit an ultrahigh sensitivity of dFWHM/d*P* = 10.13 nm GPa^−1^.

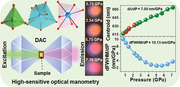

## 1 Introduction

Recently, the implementation of extreme environment to regulate the performance of materials, such as phase transition, bond length, and electronic structure, has drawn considerable interests.^[^ ^1^, ^2^ ^]^ The utilization of high mechanical pressure is widely applied in many different fields including geophysics, simulations of the processes occurring in the interior of the planets, novel material synthesis, luminescence generation, space research, and so forth.^[^ ^3^, ^4^, ^5^ ^]^ At present, the symmetric diamond anvil cell (DAC) has been intensively adopted to produce static high pressure in scientific research so as to simultaneously realize spectroscopic and structural characterization at elevated pressure.^[^ ^6^, ^7^ ^]^ In general, the pressure‐sensitive luminescent materials doped with rare‐earth or transition metal ions are often utilized to detect pressure changes in the DAC assembly through analyzing their pressure‐dependent spectral features (manometric parameters), such as full width at half maximum (FWHM), emission band position, luminescence intensity ratio, and emission decay time.^[^ ^8^, ^9^, ^10^, ^11^ ^]^ Currently, the Al_2_O_3_:Cr^3+^ (ruby) and SrB_4_O_7_:Sm^2+^ compounds are the most commonly used luminescent materials to calibrate high pressure, in which their pressure sensitivities are 0.365 and 0.255 nm GPa^−1^, respectively.^[^ ^12^, ^13^ ^]^ However, their small sensitivities cannot satisfy the growing needs of technological and scientific progress. To address this issue, diverse optical manometers based on luminescent materials, such as BaLi_2_Al_2_Si_2_N_6_:Eu^2+^ (d*λ*/d*P* = 1.58 nm GPa^−1^), Na_3_CsMg_7_(PO_4_)_6_:Eu^2+^ (d*λ*/d*P* = 2.13 nm GPa^−1^), Mg_2_Gd_8_(SiO_4_)_6_O_2_:Ce^3+^ (d*λ*/d*P* = 1.8453 nm GPa^−1^), Ca_9_NaZn(PO_4_)_7_:Eu^2+^ (d*λ*/d*P* = 5.21 nm GPa^−1^), and ZnS/CaZnOS:Mn^2+^ (d*λ*/d*P* = 6.20 nm GPa^−1^), were developed recently by researchers.^[^ ^14^, ^15^, ^16^, ^17^, ^18^ ^]^ Although the pressure sensitivity has been enhanced by searching for different luminescent materials, more attention is still needed to be applied to heighten the pressure sensing capacities of luminescent materials so as to realize the vivid application. Furthermore, the majority of previously developed optical manometers are only appropriate for examining ultra‐high pressure (i.e., *P* > 10 GPa), and the accurate measurement of relatively low pressure region (<10 GPa), in which organic and soft materials may experience phase transition, is still insufficient.^[^ ^4^ ^]^ Thereby, developing new luminescent manometers, which are available for the aforementioned relatively low‐pressure region, with fast response and high precision is of utmost importance.

Nowadays, the interest in the luminescent materials doped with transition metal ions (i.e., Bi^3+^, Mn^2+^, Mn^4+^, Fe^3+^, Cr^3+^, etc.) is boosting on account of their features of low cost, abundant resources, splendid spectral properties, and so on.^[^ ^19^, ^20^, ^21^ ^]^ Among them, Mn^2+^ with 3d^5^ electron configuration has been extensively studied as an activator due to its unique emissions arising from the ^4^T_1_(^4^G) → ^6^A_1_ spin forbidden transition.^[^ ^22^, ^23^ ^]^ Since five d‐electrons are located in the partially filled 3d shell, the crystal field of the host compounds plays an important role in affecting their optical features, that is, Mn^2+^‐activated materials of the high spin configuration tend to generate green emission when the Mn^2+^ ions occupy a site with low crystal field strength, while orange–red emission is generated when it locates at the site with high crystal field strength.^[^ ^1^, ^24^ ^]^ It is well‐known, that upon high pressure the distance between atoms is shortened, which is beneficial for improving the overlap of the adjacent orbitals, contributing to the enhanced crystal field strength.^[^ ^25^, ^26^ ^]^ Thus, the spectral characterizations of Mn^2+^‐activated luminescent materials can be adjusted via employing high pressure, which allows them to be used in the field of optical pressure sensing. On the other hand, aside from high pressure sensitivity, to ensure the designed luminescent compounds are good candidates for optical manometry, they are also required to show favorable luminescence properties, that is, high thermal stability, intense luminescence intensity, appropriate excitation, and emission wavelengths. Considering the fact that the luminescence features of Mn^2+^ are highly dependent on the chemical environment of host materials,^[^ ^27^, ^28^ ^]^ the selection of a proper host is the simplest strategy to fulfill this goal. In recent years, silicate oxyapatites, which exhibit the general chemical formula of M_10_(SiO_4_)O_6_ (M = Na, Li, K, La, Gd, Yb, Ba, Ca, etc.), have been largely developed as host compounds for rare‐earth and transition metal ions, because of their high rigidity, superior stability, unique spatial crystal structure, and ecofriendly characterizations.^[^ ^29^, ^30^, ^31^ ^]^ Among them, NaY_9_(SiO_4_)_6_O_2_ has received a lot of attention, and different types of luminescent materials, such as NaY_9_(SiO_4_)_6_O_2_:Ln^3+^/Yb^3+^ (Ln = Tm, Ho), NaY_9_(SiO_4_)_6_O_2_:Sm^3+^, NaY_9_(SiO_4_)_6_O_2_:Bi^3+^, and NaY_9_(SiO_4_)_6_O_2_:Ce^3+^/Eu^3+^,^[^ ^32^, ^33^, ^34^, ^35^ ^]^ were reported to realize various applications. Despite these, the investigation of the effect of pressure on the spectral properties of NaY_9−_ *_x_* (SiO_4_)_6_O_2_:*x*Mn^2+^ (NaY_9_(SiO_4_)_6_O_2_:*x*Mn^2+^) compounds has not been performed yet.

Here, we successfully synthesized a series of NaY_9_(SiO_4_)_6_O_2_:*x*Mn^2+^ phosphors exhibiting strong red emissions. The influence of Mn^2+^ doping on the phase purity, morphology, and luminescence characterizations of the resultant phosphors was systematically analyzed. The pressure‐dependent Raman spectra demonstrate that the phase transition does not take place in the examined pressure range. Moreover, according to the pressure‐caused huge red‐shift of the emission band and significant changes in its FWHM, it turned out that the designed optical manometers exhibit appealing pressure sensing abilities, that is, pressure sensitivities d*λ*/d*P =* 7.00 nm GPa^−1^ and d(FWHM)/d*P* = 10.13 nm GPa^−1^. The achieved results confirm that the designed phosphors have great application potential for ultra‐sensitive pressure sensing purposes, that is, modern optical manometry.

## 2 Results and Discussion

### 2.1 Structural Properties at Ambient Conditions

**Figure 1:**
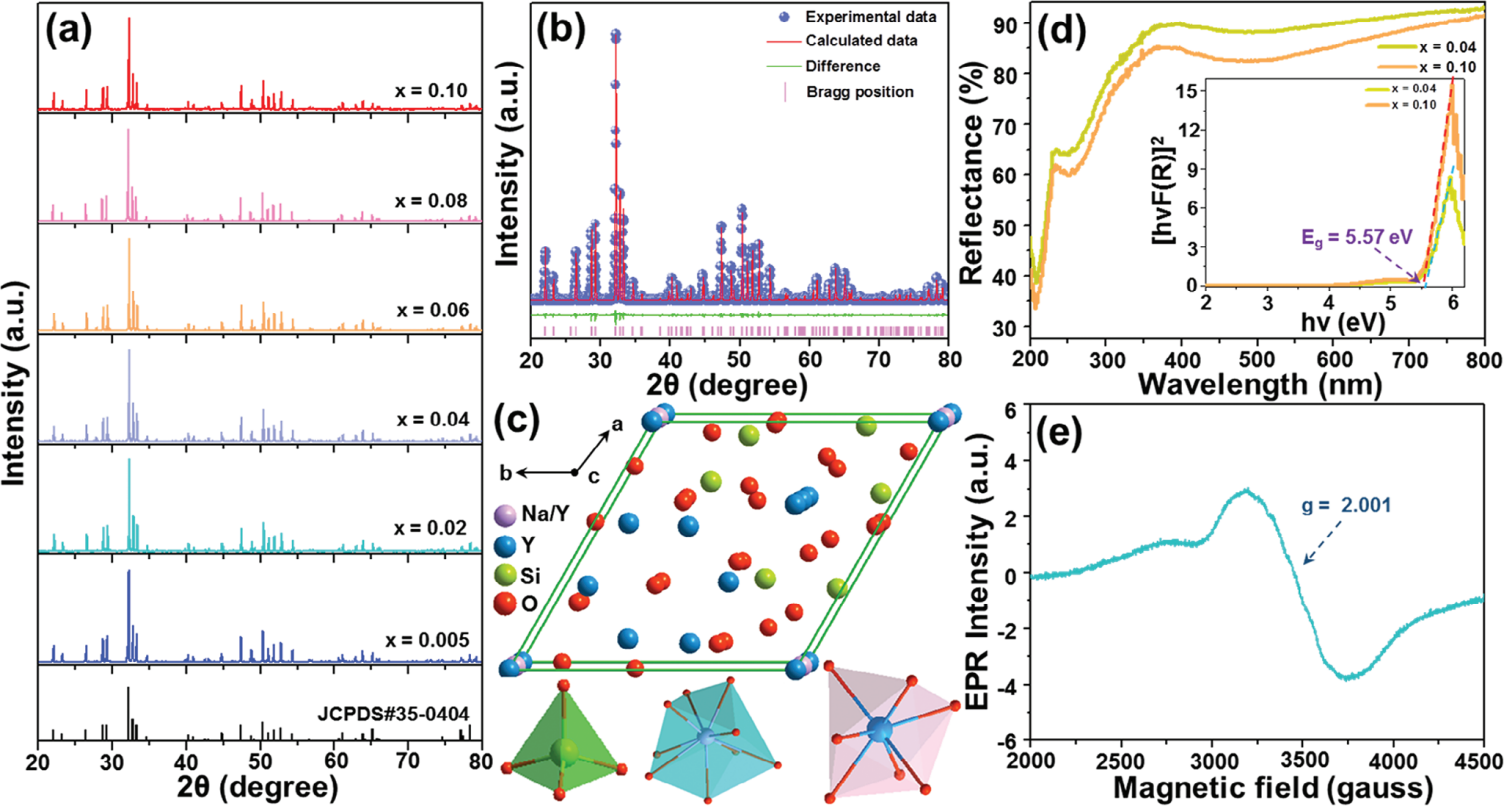
a) Powder XRD patterns of the synthesized NaY_9_(SiO_4_)_6_O_2_:*x*Mn^2+^ phosphors. Rietveld XRD refinement for the NaY_9_(SiO_4_)_6_O_2_:*x*Mn^2+^ (*x* = 0.08) sample. c) 3D representation of the crystal structure of the NaY_9_(SiO_4_)_6_O_2_ and its unit cell. d) UV–vis diffuse reflectance spectra of the NaY_9_(SiO_4_)_6_O_2_:*x*Mn^2+^ (*x* = 0.04 and 0.10) phosphors. e) EPR spectrum of the NaY_9_(SiO_4_)_6_O_2_:0.08Mn^2+^ phosphor.

**Figure** **1**a displays the powder X‐ray diffraction (XRD) profiles of the resultant phosphors to explore the impact of Mn^2+^ doping on their phase purity. As displayed, the recorded there is no significant difference between the presented diffraction patterns and they are in good accordance with the reference data for the hexagonal NaY_9_(SiO_4_)_6_O_2_ (JCPDS#35‐0404), implying that the doping of Mn^2+^ negligibly alters the crystal structure of the final compounds. To further clarify the phase composition of studied samples, the Rietveld XRD refinement was performed and the exemplary for the NaY_9_(SiO_4_)_6_O_2_:0.08Mn^2+^ phosphor and the corresponding results are shown in Figure 1b and in Table S1 (Supporting Information). Evidently, a very good correlation between the experimental data and the calculated results can be found (see Figure 1b), revealing the pure hexagonal phase of the synthesized samples. Moreover, the lattice parameters (*a*, *b*, *c*, and *V*) of the NaY_9_(SiO_4_)_6_O_2_:0.08Mn^2+^ phosphor are smaller than those for the reference NaY_9_(SiO_4_)_6_O_2_ (JCPDS#35‐0404), as listed in Table S1 (Supporting Information). This is due to the lattice contraction upon Mn^2+^ doping, that is, substituting the Y^3+^ ions of larger ionic radius with smaller Mn^2+^ ones (see Table S2, Supporting Information). As mentioned above, the Mn^2+^‐doped NaY_9_(SiO_4_)_6_O_2_ pertains to apatite‐type materials and its crystal structure is presented in Figure 1c. Herein, there are four different cationic crystallographic sites, namely, one for Si^4+^, one for Na^+^, and two Y^3+^ sites. In particular, Si^4+^ is coordinated with four oxygen atoms, forming a tetrahedron, whereas Na^+^ is surrounded by seven oxygen atoms and forms a decahedron. Notably, Y^3+^ exhibits two types of cationic sites, where I) locates at 4f site, which is surrounded by nine oxygen atoms, with C_3_ symmetry and forms tetrakaidecahedron; and II) occupies the 6 h site, which is coordinated with seven oxygen atoms, with C_s_ symmetry and forms decahedron, as depicted in Figure 1c. Herein, Mn^2+^ prefers to substitute the Y^3+^ sites, resulting in two diverse emitting centers, which will be analyzed in detail in the following part.

The exemplary UV–vis diffuse reflectance spectra of the NaY_9_(SiO_4_)_6_:O_2_:*x*Mn^2+^ phosphors with *x* = 0.04 and 0.10 are presented in Figure 1d. As displayed, the recorded UV–vis diffuse reflectance spectra can be divided into three parts, namely, I) the first absorption band in the range of 200–250 nm arises from the absorption of host, II) the second band ranging from 250 to 400 nm is assigned to the O^2−^ → Mn^2+^ charge transfer process, and III) the third broad band (400–600 nm) comes from the d–d transition of Mn^2+^.^[^ ^21^ ^]^ Besides, the optical band gaps (*E*_g_) of the studied samples were also evaluated, as defined below^[^ ^25^, ^36^ ^]^

(1) $$F\left(R\right)={\left(1-R\right)}^{2}/2R$$

(2) $$hvF\left(R\right)=A{\left(hv-E_{g}\right)}^{n}$$

where *R*, *hv*, and *A* are attributed to the reflection coefficient, photon energy, and constant, respectively, and the *n* value is determined by the category of semiconductor. Here, *n* is 1/2 since NaY_9_(SiO_4_)_6_O_2_ is a direct semiconductor.^[^ ^35^ ^]^ Accordingly, the determined *E*_g_ value of the prepared compounds is around 5.57 eV, and it is almost unchanged by tuning Mn^2+^ content (see inset in Figure 1d). On the other hand, the X‐ray photoelectron spectroscopy (XPS) results shown in Figure S1 (Supporting Information) confirm that the elements Na, Y, Si, O, and Mn are present and uniformly distributed in the synthesized phosphors (for details, see the Supporting Information). Furthermore, the electron paramagnetic resonance (EPR) analysis was also performed to get a deeper insight into the information of Mn^2+^, as displayed in Figure 1e. In general, the EPR spectrum of Mn^2+^ is a six‐hyperfine structure caused by the interaction of its nuclear and electron spins.^[^ ^37^, ^38^ ^]^ Unfortunately, the recorded EPR spectrum is significantly broadened, has *g* value of about 2.00, as demonstrated in Figure 1e, which is associated with the intense magnetic dipolar interactions among Mn^2+^ ions.^[^ ^37^ ^]^ The similar phenomenon was also reported in other Mn^2+^‐activated phosphors, in which the high Mn^2+^ content will promote the spin‐relaxation process and lead to the broadening of the EPR spectrum.^[^ ^37^, ^38^ ^]^

**Figure 2:**
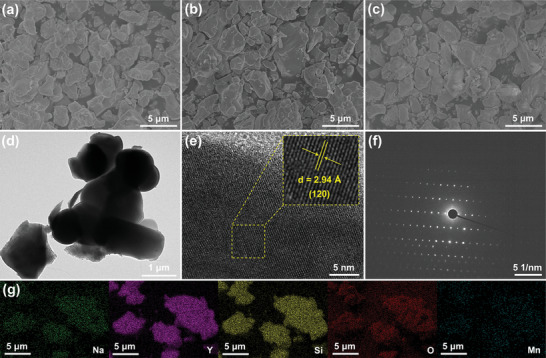
SEM images of the a) NaY_9_(SiO_4_)_6_O_2_:0.005Mn^2+^, b) NaY_9_(SiO_4_)_6_O_2_:0.08Mn^2+^, and NaY_9_(SiO_4_)_6_O_2_:0.10Mn^2+^ phosphors. d) TEM image, e) high‐resolution TEM micrograph, f) SAED pattern, and g) elemental mapping results for the NaY_9_(SiO_4_)_6_O_2_:0.08Mn^2+^ phosphor.

The morphological features of the final products were examined by employing the scanning electron microscopy (SEM) and transmission electron microscopy (TEM). As displayed in the SEM graphs in **Figure** **2**a–c and Figure S2 (Supporting Information), the synthesized materials form microparticles with irregular shape and size. Moreover, the average particle sizes of the studied compounds are be 2.01, 2.09, 2.07, 2.06, 2.01, and 2.08 µm, respectively, when *x* value is 0.005, 0.02, 0.04, 0.06, 0.08, and 0.10, as illustrated in Figure S3 (Supporting Information). These results suggest that morphological characterizations of final products are independent of the Mn^2+^ content. Moreover, the TEM image shown in Figure 2d also indicates that the inhomogeneous microparticles exist in the resulting phosphors. The high‐resolution TEM image contains recognizable lattice fringes, with *d*‐spacing of around 2.94 Å, corresponding to the (120) plane of the hexagonal NaY_9_(SiO_4_)_6_O_2_ (JCPDS#35‐0404), as shown in Figure 2e. Besides, the bright dots, which are observed in the selected area electron diffraction (SAED) pattern in Figure 2f, manifest the single‐crystalline nature of the designed compounds. Additionally, the constituting elements (i.e., Na, Y, Si, O, and Mn) in synthesized phosphors are uniformly distributed over the entire microparticles.

### 2.2 Luminescence Properties at Ambient Conditions

**Figure 3:**
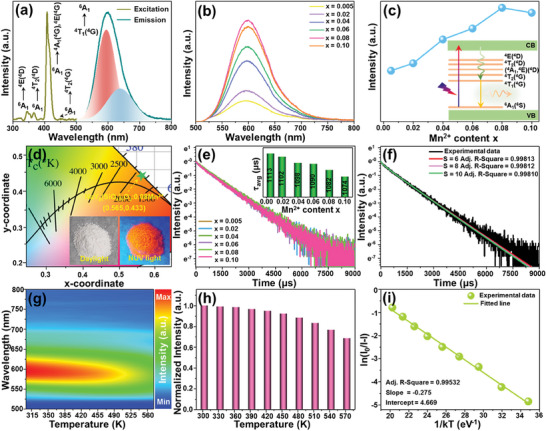
a) Excitation and emission spectra of the NaY_9_(SiO_4_)_6_O_2_:0.08Mn^2+^ phosphor. b) Emission spectra of the NaY_9_(SiO_4_)_6_O_2_:*x*Mn^2+^ (0.005 ≤ *x* ≤ 0.10) phosphors. c) Dependence of emission intensity on Mn^2+^ content. d) CIE chromaticity diagram for the NaY_9_(SiO_4_)_6_O_2_:0.08Mn^2+^ phosphor and the inset shows the corresponding optical images. e) The room temperature luminescence decay curves for the NaY_9_(SiO_4_)_6_O_2_:*x*Mn^2+^ (0.005 ≤ *x* ≤ 0.10) phosphors. f) Fitted decay curve of the NaY_9_(SiO_4_)_6_O_2_:0.08Mn^2+^ phosphor using I–H model. g) Emission spectra of the NaY_9_(SiO_4_)_6_O_2_:0.08Mn^2+^ phosphor at elevated temperature. h) Temperature‐dependent emission intensity. i) Plot of ln(I/I_0_‐1) versus 1/*kT* for the NaY_9_(SiO_4_)_6_O_2_:0.08Mn^2+^ phosphor.

The excitation spectrum of the NaY_9_(SiO_4_)_6_O_2_:0.08Mn^2+^ phosphor was monitored at 598 nm and shown in **Figure** **3**a. As demonstrated, the excitation profile consists of four bands at about 345, 366, 408, and 454 nm, corresponding to the d–d transitions of Mn^2+^, namely, from ^6^A_1_ ground state to ^4^E(^4^D), ^4^T_2_(^4^D), ^4^A_1_(^4^G)/^4^E(^4^G), and ^4^T_2_(^4^G) excited states, respectively.^[^ ^38^, ^39^ ^]^ Note, the excitation band at 408 nm has the strongest intensity, and thus, it is selected as the optical excitation wavelength. The emission spectrum of the NaY_9_(SiO_4_)_6_O_2_:0.08Mn^2+^ phosphor (*λ*_ex_ = 408 nm), shown in Figure 3a, is inhomogeneously broadened and can be deconvoluted into two Gaussian components with the maxima at about 594 and 638 nm, implying that the Mn^2+^ ions occupy two crystallographic positions in the studied samples. This result coincides well with the deduction obtained from the structural analysis. It is well‐known, that the crystal field splitting strength (*D*_q_) of the activator ion has a decisive role and affects the spectral position of the emission band, and the *D*_q_ value can be achieved via the following expression^[^ ^39^, ^40^ ^]^

(3) $$D_{q}=\frac{ze^{2}r^{4}}{6R^{5}}\;$$

where the average bond distance is denoted by *R*, *Z* is associated with the valence of the anion, the radius of the d‐electron wave function is represented by *r*, and *e* pertains to the charge of electron. Evidently, the *Z*, *e*, and *r* values are fixed, and thus, *D*_q_ is inversely proportional to *R* ^5^. In the present work, when Y^3+^ occupies the 4f site, the average *R* value of Y─O is around 2.534 Å, whereas it decreases to about 2.437 Å when Y^3+^ takes up 6 h site. Thereby, it is reasonable to deduce that the Mn^2+^ locates at ninefold coordinated Y^3+^ site, corresponding to the emission at 638 nm, while the emission at 594 nm is assigned to the d–d transition of Mn^2+^ occupying the sevenfold coordinated Y^3+^ site.

The emission spectra (*λ*_ex_ = 408 nm) of the resulting phosphors with different Mn^2+^ concentrations were measured in order to optimize the content of Mn^2+^ in the NaY_9_(SiO_4_)_6_O_2_ host lattice, as shown in Figure 3b. Apparently, all of the final compounds emit the featured emissions of Mn^2+^ and their intensities are affected by dopant content. As *x* value increases, the fluorescence intensity increases gradually and reaches the highest value when *x* = 0.08 (see Figure 3c), whereas the concentration quenching takes place when *x* > 0.08. To comprehend the concentration quenching mechanism in depth, the critical distance (*R*_c_) was analyzed according to the following expression^[^ ^41^ ^]^

(4) $$R_{c}=\;2{\left[\frac{3V}{4\pi x_{c}Z}\right]}^{1/3}$$

where the cell volume, the amount of cations in a unit cell and critical doping concentration are denoted by *V*, *Z*, and *x*_c_, respectively, and their values are 509.220 Å^3^, 1, and 0.0089, respectively. Thus, the *R*_c_ value of Mn^2+^ in NaY_9_(SiO_4_)_6_O_2_ host lattices is estimated to be about 70.02 Å, which is more than 5 Å, implying that the concentration quenching mechanism results from the electric multipolar interaction.^[^ ^42^ ^]^ The occurring luminescence process is depicted by the energy level diagram of Mn^2+^, as shown in the inset of Figure 3c. Furthermore, upon near‐UV light irradiation, visible red emission is detected in the prepared samples, and the color coordinates for the emitted light by the sample with the optimal Mn^2+^ concentration are (0.565,0.433), as presented in Figure 3d.

Figure 3e depicts the room temperature emission decay curves of the NaY_9_(SiO_4_)_6_O_2_:*x*Mn^2+^ phosphors, in which the excitation and monitoring wavelengths were 408 and 598 nm, respectively. As presented, the recorded decay curves are able to be fitted via a second‐order exponential expression, which further confirms the presence of two emitting centers in the designed phosphors,^[^ ^39^ ^]^ as defined below

(5) $$I\;\left(t\right)=I_{0}\;+A_{1}\exp\left(-t/\tau_{1}\right)+A_{2}\exp\left(-t/\tau_{2}\right)$$

where the fluorescence intensities at time *t* and *t* = 0 are denoted as *I*(*t*) and *I*_0_, respectively, *τ_i_* (*i* = 1, 2) is the emission decay times and *A_i_* (*i* = 1, 2) refers to amplitude. Furthermore, the average decay time (*τ*_avg_) is evaluated through the following formula

(6) $$\tau_{\mathrm{avg}}=\frac{A_{1}\tau_{1}^{2}+A_{2}\tau_{2}^{2}}{A_{1}\tau_{1}+A_{2}\tau_{2}}\;$$

Evidently, as the *x* value increases, the *τ*_avg_ is shortened, namely, changes from 1113 to 1074 µs (see inset of Figure 3e). The shortened decay time, which is caused by the enhanced non‐radiative relaxation processes, also confirms the existence of a concentration quenching process in the final compounds, and its mechanism can be verified through employing the Inokuti–Hirayama (I–H) theoretical model equation, as presented below.^[^ ^25^, ^43^ ^]^

(7) $$I\;\left(t\right)=I_{0}\;\exp\left[-\frac{t}{\tau_{0}}-\alpha {\left(\frac{t}{\tau_{0}}\right)}^{3/s}\right]$$

where the intrinsic decay time of Mn^2+^ is demonstrated by *τ*_0_, *α* is a constant, whereas *s* can have three different values of 6, 8, and 10, which correspond to dipole–dipole, dipole–quadrupole, and quadrupole–quadrupole interactions, respectively. From Figure 3f, it is evident that the best fitting result is realized when *s* = 6, implying that the electric dipole–dipole interaction mainly contributes to the interionic interaction mechanism of Mn^2+^ in the developed materials.

In order to satisfy the practical applications, the luminescent materials are expected to simultaneously exhibit good luminescence behaviors and admirable thermal stability properties. The temperature‐related emission spectra of the NaY_9_(SiO_4_)_6_O_2_:0.08Mn^2+^ phosphors measured in the range of 303–573 K are displayed in Figure 3g. With temperature elevation, the emission intensity declines due to the enhanced non‐radiative transitions at high temperature. Surprisingly, the resulting phosphors display excellent thermal stability, in which the emission intensity at 423 K keeps around 95% of its initial value at 303 K (see Figure 3h). Besides, by the use of the following function, the corresponding activation energy (Δ*E*) is evaluated^[^ ^44^, ^45^ ^]^

(8) $$I\;\left(T\right)=\frac{I_{0}}{1+A\exp\left(-\Delta E/kT\right)}\;$$

where the initial emission intensity is labeled as *I*_0_, the emission intensity at time *t* is denoted by *I*(*T*), *A* is a constant, and *k* refers to Boltzmann coefficient, where its value is 8.629 × 10^−5^ eV K^−1^. Through studying the experimental data by Equation (8), the Δ*E* value for Mn^2+^ in the synthesized phosphors is calculated to be around 0.275 eV (see Figure 3i). This result indicates that the resulting compounds with splendid luminescence properties and high thermal stability are suitable for practical applications, such as solid‐state lighting, optical manometry, and so forth.

### 2.3 Structural Properties at High‐Pressure Conditions

**Figure 4:**
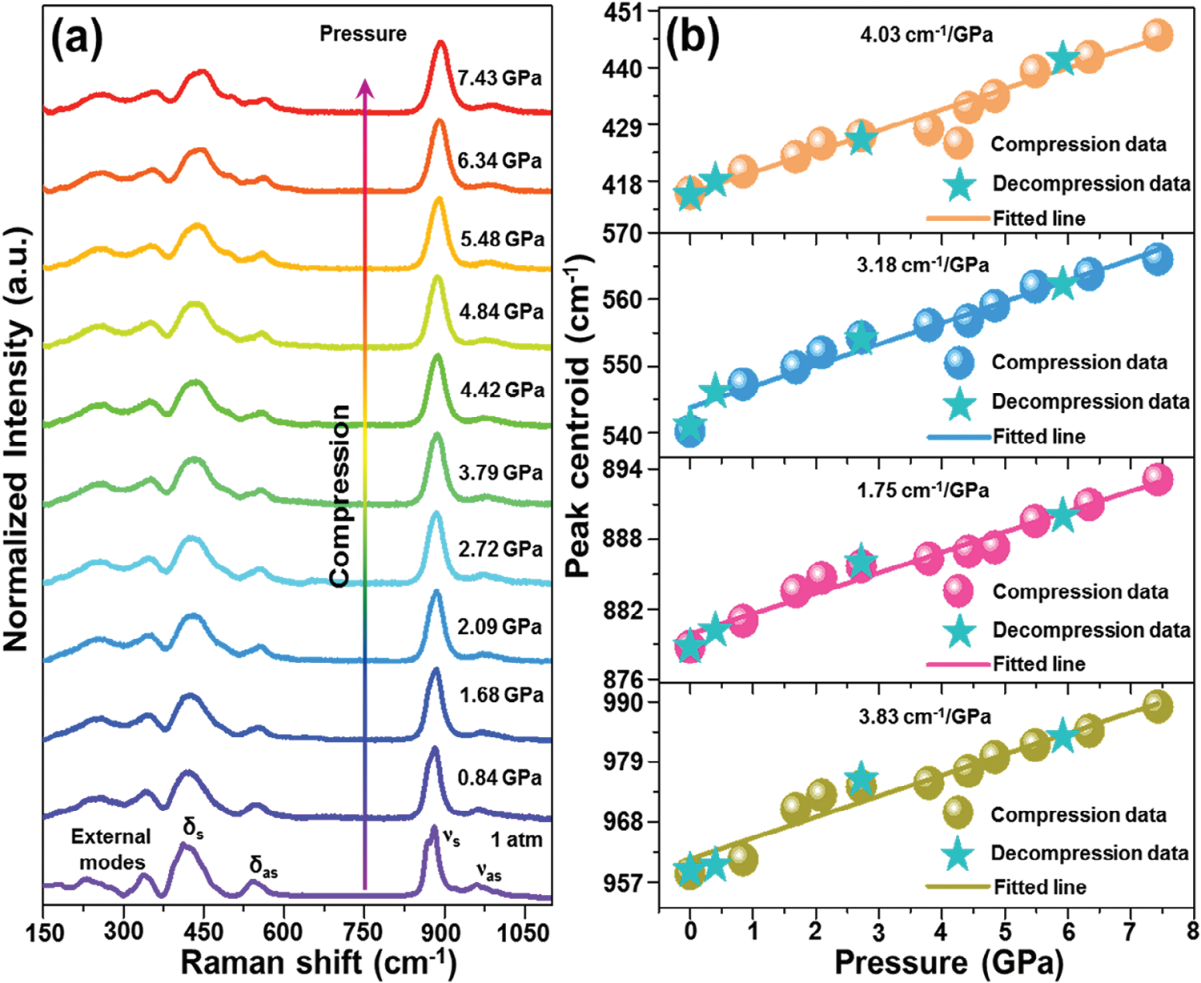
a) Raman spectra and b) the determined peak centroids for the NaY_9_(SiO_4_)_6_O_2_:0.08Mn^2+^ phosphor as a function of pressure.

By investigating the pressure‐dependent Raman spectra, the structural stability of the designed phosphors at high pressure is explored. At ambient pressure, the recorded Raman spectrum of the NaY_9_(SiO_4_)_6_O_2_:0.08Mn^2+^ phosphor can be divided into two parts, that is, below and above 370 cm^−1^, as illustrated in **Figure** **4**a. Of these, the Raman modes in the range of 370–1100 cm^−1^ all pertain to the internal modes of the pseudo‐tetrahedral silicate groups in the apatite‐type compounds.^[^ ^46^, ^47^ ^]^ In particular, the relatively weak band at about 958 cm^−1^ results from the asymmetric stretching mode *v*_as_, the strongest band at 879 cm^−1^ is assigned to the symmetric stretching mode versus, the band located at ≈540 cm^−1^ corresponds to the asymmetric bending silicate group mode *δ*_as_, and the peak at around 416 cm^−1^ originates from the symmetric bending mode *δ*_s_.^[^ ^46^, ^47^ ^]^ Moreover, the bands below 370 cm^−1^ are attributed to the external modes, which may be caused by the translations and rotational oscillations of the silicate group, Na and Y units.^[^ ^46^, ^47^ ^]^ Notably, as pressure increases (i.e., from 1 atm to 7.43 GPa), the aforementioned Raman modes do not disappear and there are no any extra peaks observed in the Raman spectra (see Figure 4a), implying that the external pressure does not alter the phase structure of studied phosphors, at least in the investigated pressure range. Furthermore, although the Raman bands are not vanished, they shift to higher wavenumbers (higher energies) during the compression process, as depicted in Figure 4b, which is associated with the bond shortening at high pressure. In addition, through linearly fitting, it is found that the shift rates of these Raman peaks initially centered at 416, 540, 879, and 958 cm^−1^ are 4.03, 3.18, 1.75, and 3.83 cm^−1^ GPa^−1^, respectively. Importantly, the structure of the studied material can come back to its original state when it undergoes the decompression process, as presented in Figure S4 (Supporting Information), showing the Raman spectra measured during the pressure release cycle. These results demonstrate that the developed luminescent materials are structurally stable at elevated pressure, and their compression is fully reversible, which is beneficial for applications in optical manometry.

### 2.4 Luminescence Properties at High‐Pressure Conditions

**Figure 5:**
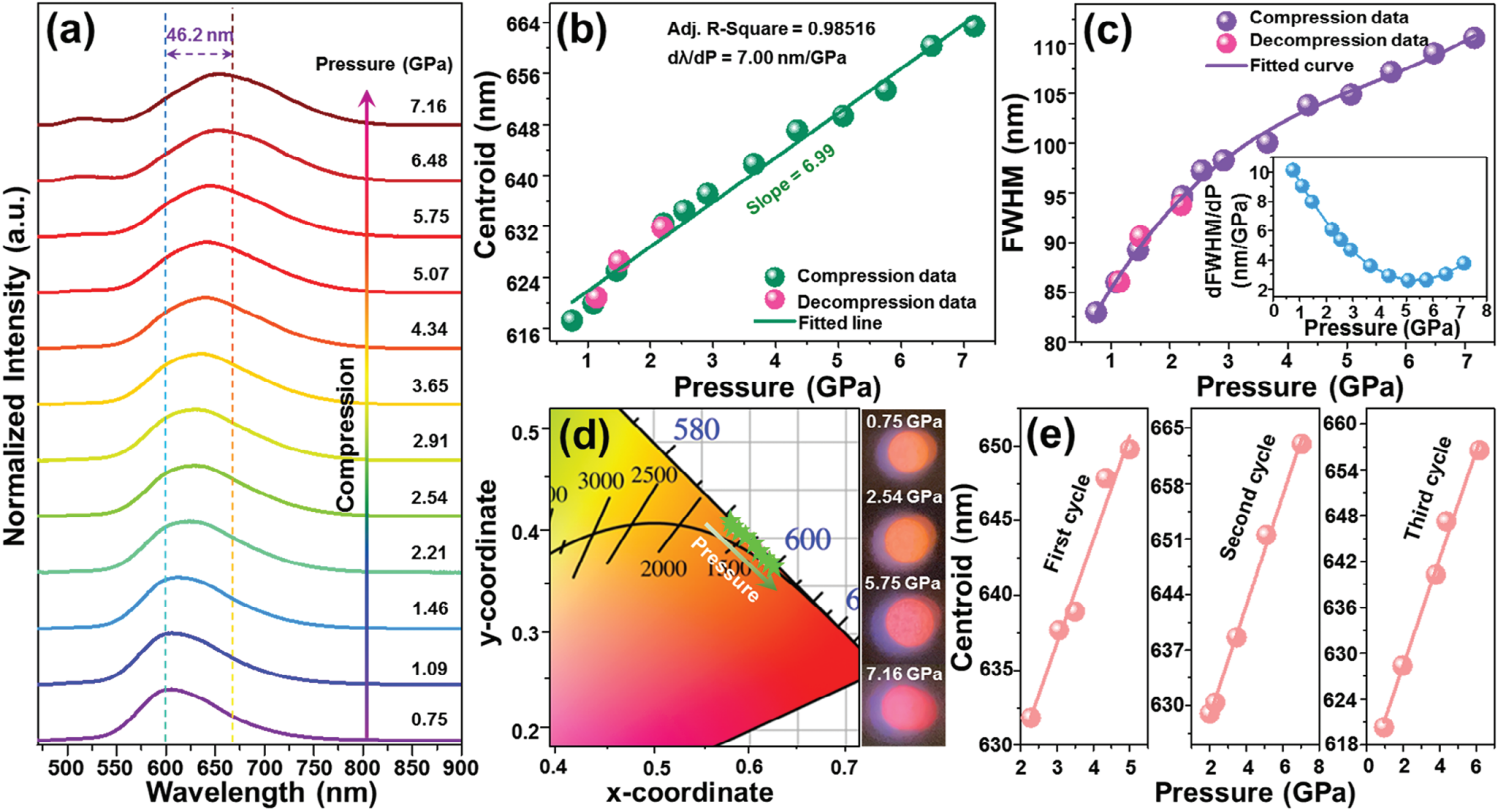
a) Normalized emission spectra of the NaY_9_(SiO_4_)_6_O_2_:0.08Mn^2+^ phosphor at diverse pressures. Calculated b) emission band centroid, c) FWHM, and d) 1931 CIE chromaticity coordinates for the NaY_9_(SiO_4_)_6_O_2_:0.08Mn^2+^ phosphor as a function of pressure. Inset of (c) illustrates pressure dependent the d(FWHM)/d*P* values. e) Cycling experiments, illustrating Mn^2+^ emission band centroid at various pressures, within three compression–decompression cycles.

For the purpose of examining the feasibility of the developed compounds working as optical manometers, the emission spectra of the NaY_9_(SiO_4_)_6_O_2_:0.08Mn^2+^ material at various isostatic pressure values (i.e. 0.75 ≤ *P* ≤ 7.16 GPa) were measured upon 375 nm excitation, as shown in **Figure** **5**a. In this work, the experiments were carried out in a high‐pressure DAC and Al_2_O_3_:Cr^3+^ was used as a pressure indicator. Evidently, as pressure increases, the luminescence profiles still consist of a single emitting band, whereas the featured emission band of Mn^2+^ (i.e., ^4^T_1_(^4^G) → ^6^A_1_ transition) is shifted to longer wavelengths, that is, its centroid wavelength gradually increases from 617.2 to 663.4 nm. This phenomenon is mainly associated with the increased crystal‐field strength (enhancement of the splitting of multiplets in the contracted structure), but also it is related to the enhanced nephelauxetic effect.^[^ ^3^, ^16^ ^]^ From Equation (3), one knows that the *D*_q_ value is determined by crystal‐field strength, so it is related to the bond distance, that is

(9) $$D_{q}\propto 1/R^{5}$$

With raising the pressure, the lattice contraction will take place, resulting in the shortening of bond length, which results in a higher *D*_q_ value, leading to lowering of the energy of the emitting state according to the Tanabe Sugano diagram for the d^5^ electronic configuration, which results in the red‐shift of the emission band.^[^ ^16^ ^]^ On the other hand, it is well‐known, that the distance of the Mn^2+^─O^2−^ bond will be shortened during the compression process and its covalency will be enhanced, resulting in the deteriorated free‐ion (coulombic and spin orbit) coefficients, and thus, the energy difference between the excited level and ground state is decreased, resulting in the overall red‐shift of the emission band at high pressure.^[^ ^25^ ^]^ The superposition of these factors, results in the observed monotonic, pressure‐triggered spectral red‐shift in the studied phosphor material. Furthermore, it is shown in Figure 5b that the relation between pressure and emission band centroid (*λ*) is linear, in which it can be described as *λ* = 7.00*P* + 614.85. Thereby, the pressure sensitivity (d*λ/*d*P*), which is assigned to the shift rate of emission band centroid with pressure, is almost 7.00 nm GPa^−1^. Notably, the obtained d*λ/*d*P* value is not only 19 times larger than the widely used Al_2_O_3_:Cr^3+^ (d*λ/*d*P =* 0.365 nm GPa^−1^), but also larger than most of the recently reported optical pressure sensor, as listed in **Table** **1**. Moreover, when the studied samples undergo the decompression process, the emission band returns to its initial position, as demonstrated in Figure 5b, confirming its good stability and reversibility under extreme conditions. These results suggest that the high‐sensitive contactless optical manometry can be realized by monitoring the shift of the emission band centroid of Mn^2+^‐activated NaY_9_(SiO_4_)_6_O_2_ red‐emitting phosphors.

**Table 1:** Comparative analysis of pressure sensing capabilities in different luminescent materials emitting in the visible range utilized for optical manometry.

Luminescent materials	*λ*_ex_ [nm]	Sensing parameter	Sensitivity [nm GPa^−1^]	Reference
Lu_2_Mg_2_Al_2_Si_2_O_12_:Eu^2+^/Mn^2+^	355	Emission band	3.53	[1]
Sr_8_Si_4_O_12_Cl_8_:Eu^2+^	280	Emission band	9.69	[3]
Y_6_Ba_4_(SiO_4_)_6_F_2_:Ce^3+^	445	FWHM	1.81	[9]
Sr_2_LuNbO_6_:Mn^4+^	532	Emission band	0.82	[11]
BaLi_2_Al_2_Si_2_N_6_:Eu^2+^	355	Emission band	1.58	[14]
Mg_2_Gd_8_(SiO_4_)_6_O_2_:Ce^3+^/Mn^2+^	355	Emission band	1.84	[16]
Ca_9_NaZn(PO_4_):Eu^2+^	355	Emission band	5.21	[17]
Ca_2_Gd_8_Si_6_O_26_:Ce^3+^	300	FWHM	2.45	[25]
Ca_4_Y_3_Si_7_O_15_N_5_:Eu^2+^	355	Emission band	1.13	[26]
NaY_9_(SiO_4_)_6_O_2_:Mn^2+^	375	Emission band	7.00	This work
NaY_9_(SiO_4_)_6_O_2_:Mn^2+^	375	FWHM	10.13	This work

Aside from the red‐shift of the emission band, the FWHM of the detected emission band of Mn^2+^ also depends on pressure. As pressure raises, the FWHM of the Mn^2+^ emission band significantly increases from 83.0 to 110.6 nm, and the relation between pressure and FWHM obeys a third order polynomial function, namely, FWHM = 74.37*P* ^3^ + 12.81*P* ^2^ − 1.92*P* + 0.12. Moreover, according to the definition of pressure sensitive based on FWHM, namely, dFWHM/d*P*, the pressure dependent dFWHM/d*P* value is plotted and shown in the inset of Figure 5c. Clearly, the maximum dFWHM/d*P* value is found to be as high as 10.13 nm GPa^−1^ in the relatively low pressure range. Compared with other FWHM‐based optical manometers based, the obtained pressure sensitivity of the developed sensor is the highest, as displayed in Table 1, implying its great application potential in optical pressure sensing. Besides, the impact of pressure on the emitting color (visual sensing) of the Mn^2+^‐doped NaY_9_(SiO_4_)_6_O_2_ phosphor was also studied, and the corresponding results are illustrated in Figure 5d. With elevating pressure in the range of 0.75–7.16 GPa, the color coordinates of the NaY_9_(SiO_4_)_6_O_2_:0.08Mn^2+^ phosphor gradually change from (0.584,0.413) to (0.618,0.382), as listed in Table S3 (Supporting Information), and it all locate in red region, which is further clarified by the recorded optical images (see Figure 5d). Aside from the changed color coordinate, the color correlated temperature of the resultant phosphors also alters from 2504 to 2424 K as pressure rises, as displayed in Table S3 (Supporting Information) (for details, see Supporting Information). In addition, the cycling experiments were also performed to probe the stability of developed phosphors and their reliability as manometers. Fortunately, the red‐shift of the emission band centroid remains fully reversible even it undergoes three compression–decompression cycles (Figure 5e), demonstrating that the synthesized luminescent materials have splendid stability and reversibility under extreme conditions of pressure. These achievements manifest that the pressure‐triggered remarkable changes of the determined manometric parameters (i.e., peak centroid and FWHM) in the Mn^2+^‐activated NaY_9_(SiO_4_)_6_O_2_ red‐emitting phosphors make them promising candidates for modern, ultrasensitive optical manometry.

## 3 Conclusion

In summary, the NaY_9_(SiO_4_)_6_O_2_:*x*Mn^2+^ red‐emitting phosphors were synthesized to investigate their applicability for optical manometry, resulting in the development of the most sensitive optical manometer up to date, operating in the band‐width mode. The phase composition, morphology, elemental constituent, and luminescence behaviors have been systematically studied. Excited at 408 nm, all of the synthesized compounds emit the featured emissions of Mn^2+^, that is, that band which can be deconvoluted into two peaks caused by the different occupation sites by Mn^2+^ ions, and its intensity is determined by the dopant content. Based on numerical analysis of the experimental data, we found that the concentration quenching mechanism of Mn^2+^ in studied samples is mainly contributed by electric dipole–dipole interaction, with *R*_c_ value of 33.68 Å. Besides, the resulting phosphors have admirable thermal stability and the corresponding Δ*E* value of about 0.275 eV. On the other hand, to clarify the possible applications of the final compounds for optical manometry, the pressure‐dependent Raman and emission spectra were recorded. The Raman spectra confirm that any phase transition does not occur in the designed phosphors, in the pressure range studied, which is critical for optical sensing applications. Furthermore, as pressure elevates, both the emission band centroid and FWHM show huge and monotonic changes. Notably, via utilizing the pressure‐caused red‐shift of the emission band centroid and FWHM as sensing parameters, their maximum pressure sensitivities reach up to 7.00 and 10.13 nm GPa^−1^, respectively. It is worth noting, that the achieved great pressure sensitivity of the manometric parameter based on the FWHM, currently classifies our sensor as the most sensitive manometer reported working in this mode. Inspired by the high sensitivity and great stability at an elevated pressure of the Mn^2+^‐activated NaY_9_(SiO_4_)_6_O_2_ red‐emitting phosphors, they are promising candidates as optical pressure sensors, in order to rapidly monitor pressure changes in diverse harsh environments, such as deep sea, ultra‐heavy constructions, planetary interiors, and so forth.

## 4 Experimental Section

### Synthesis of NaY_9_(SiO_4_)_6_O_2_:xMn^2+^ Red‐Emitting Phosphors

By the utilization of a high‐temperature solid‐state reaction method, a series of NaY_9_(SiO_4_)_6_O_2_:*x*Mn^2+^ (0.005 ≤ *x* ≤ 0.10) red‐emitting phosphors were prepared. Herein, the raw materials were Na_2_CO_3_ (99.5%), Y_2_O_3_ (99.9%), SiO_2_ (99.5%), and MnCO_3_ (99.95%), and they were all bought from Aladdin Company. The proper amounts of these above powders were weighted on the basis of the stoichiometric ratio, and then, they were adequately mixed and ground in an agate mortar. Thereafter, they were sintered in a furnace, at calcination temperature of 1400 °C, heating rate was 5 °C min^−1^, for 6 h under a reduction atmosphere (N_2_/H_2_ = 95%/5%). Ultimately, when the temperature decreases to room temperature, the final products were collected for further characterization.

### Measurements and Characterization

The phase purity, elemental composition, morphology and light harvesting capacity of the studied samples were tested via a X‐ray diffractometer (Bruker D8; Cu kα radiation), XPS spectrometer (Thermo Scientific K‐Alpha), FE‐SEM (Hitachi SU‐70), TEM (JEM‐2100F, JEOL), and UV–vis spectrophotometer (Cary 5000), respectively. Using an EPR spectrometer (JES FA200), the EPR spectrum of the prepared phosphor was tested. Furthermore, the luminescence properties and decay curves of the final products were studied by a fluorescence spectrometer (Edinburgh FS5).

For the sake of measuring the Raman and emission spectra of studied compounds, a DAC with 400 µm diamond culets, which was developed in the University of Paderborn (Germany), was utilized. The resulting samples were placed in a hole (≈150 µm) drilled in a 250 µm thick stainless steel gasket. Herein, the methanol/ethanol/water = 16:3:1 solution was employed as the pressure transmission medium and the pressure calibration was performed via analyzing the spectral shift of ruby balls, that is, Al_2_O_3_:Cr^3+^ (*R*_1_ line). Excited at 375 nm, the pressure‐related emission spectra were detected by a spectrometer (Andor Shamrock 500i) attached with a silicon CCD camera detector, whereas the Raman spectra at high pressure were examined through a confocal micro‐Raman system (Renishaw InVia), in which a power controllable 532 nm laser diode was used.

## Conflict of Interest

The authors declare no conflict of interest.

## Supporting information

Supporting Information

## Data Availability

The data that support the findings of this study are available from the corresponding author upon reasonable request.
