# Supplementary material for: Pressure‐Induced Remarkable Spectral Red‐Shift in Mn2+‐Activated NaY9(SiO4)6O2 Red‐Emitting Phosphors for High‐Sensitive Optical Manometry
Source: Adv Sci (Weinh). 2023 Dec 16;11(9):2308221. doi: 10.1002/advs.202308221 (PMC10916622; doi:10.1002/advs.202308221)
Supplement: Supplementary file 1 — Supporting Information [file ADVS-11-2308221-s001.pdf]

ADVANCED SCIENCE
Open Access

Supporting Information

for *Adv. Sci.*, DOI 10.1002/advs.202308221

Pressure-Induced Remarkable Spectral Red-Shift in Mn^{2+}-Activated $NaY_9(SiO_4)_6O_2$ Red-Emitting Phosphors for High-Sensitive Optical Manometry

Qifeng Zeng, Marcin Runowski, Junpeng Xue, Laihui Luo, Lukasz Marciniak, Víctor Lavín and Peng Du**

Pressure-induced remarkable spectral red-shift in Mn^{2+}-activated $NaY_9(SiO_4)_6O_2$ red-emitting phosphors for high-sensitive optical manometry

Qifeng Zeng, Marcin Runowski, Junpeng Xue, Laihui Luo, Lukasz Marciniak, Victor Lavine, Peng Du**

Mr. Q. Zeng, Prof. L. Luo, Prof. P. Du

School of Physical Science and Technology, Ningbo University, 315211 Ningbo, Zhejiang, China

*E-mail: dupeng@nbu.edu.cn (P. Du)

Prof. M. Runowski

Adam Mickiewicz University, Faculty of Chemistry, Uniwersytetu Poznańskiego 8, 61-614 Poznań, Poland

*E-mail: runowski@amu.edu.pl (M. Runowski)

Dr. J. Xue

School of Science, Jiangsu University of Science and Technology, Zhenjiang 212100, China

Prof. L. Marciniak

Institute of Low Temperature and Structure Research, Polish Academy of Sciences, Okólna 2, 50-422 Wrocław, Poland.

Prof. V. Lavine

Departamento de Física, MALTA-Consilider Team, Universidad de La Laguna, Apartado de Correos 456, E-38200 San Cristóbal de La Laguna, Santa Cruz de Tenerife, Spain

Table S1. Lattice parameters of $NaY_9(SiO_4)_6O_2$:0.08Mn^{2+} phosphors and $NaY_9(SiO_4)_6O_2$ host (JCPDS#35-0404).

Parameter	$NaY_9(SiO_4)_6O_2$	$NaY_9(SiO_4)_6O_2$:0.08Mn^{2+}
Phase structure	hexagonal phase	hexagonal phase
$a = b$	9.335 Å	9.33375 Å
c	6.757 Å	6.74936 Å
V	509.933 $Å^3$	509.2204 $Å^3$
$\alpha = \beta$	90°	90°
γ	120°	120°
R_{wp}	-	0.3636
R_p	-	0.1972
χ^2	-	2.081

Table S2. Ionic radii of Mn^{2+} and Y^{3+} with different coordinate numbers.

Ion species	Coordinate number	
	7	9
Mn^{2+}	0.90 Å	1.045 Å
Y^{3+}	0.96 Å	1.075 Å

Table S3. Color coordinates of $NaY_9(SiO_4)_6O_2$:0.08Mn^{2+} phosphors as a function of pressure.

Pressure (GPa)	Color coordinates		CCT value
	x	*x*	
0.75	0.584	0.584	2504
1.09	0.587	0.587	2496
1.46	0.596	0.596	2474
2.21	0.605	0.605	2453
2.54	0.609	0.609	2444
2.91	0.609	0.609	2444
3.65	0.613	0.613	2435
4.34	0.617	0.617	2426
5.07	0.616	0.616	2428
5.75	0.616	0.616	2428
6.48	0.617	0.617	2426
7.16	0.618	0.618	2424

From previous literatures,[S1,S2] it is clear that the correlated color temperature (*i.e*. CCT) can be estimated from the color coordinates via employing the following expressions:

$$CCT = -437n^3 + 3601n^2 - 6846n + 5514.31 \quad \text{(S1)}$$

$$n = (x - x_e)/(y - y_e) \quad \text{(S2)}$$

where (x,y) is the color coordinate of the resultant samples and (x_e,y_e) = (0.3320,0.1858). Via using the aformentioned expressions as well as the determined color coordinates, the pressure dependent CCT values of $NaY_9(SiO_4)_6O_2$:0.08Mn^{2+} phosphor are estimated and the corresponding results are listed in Table S2.

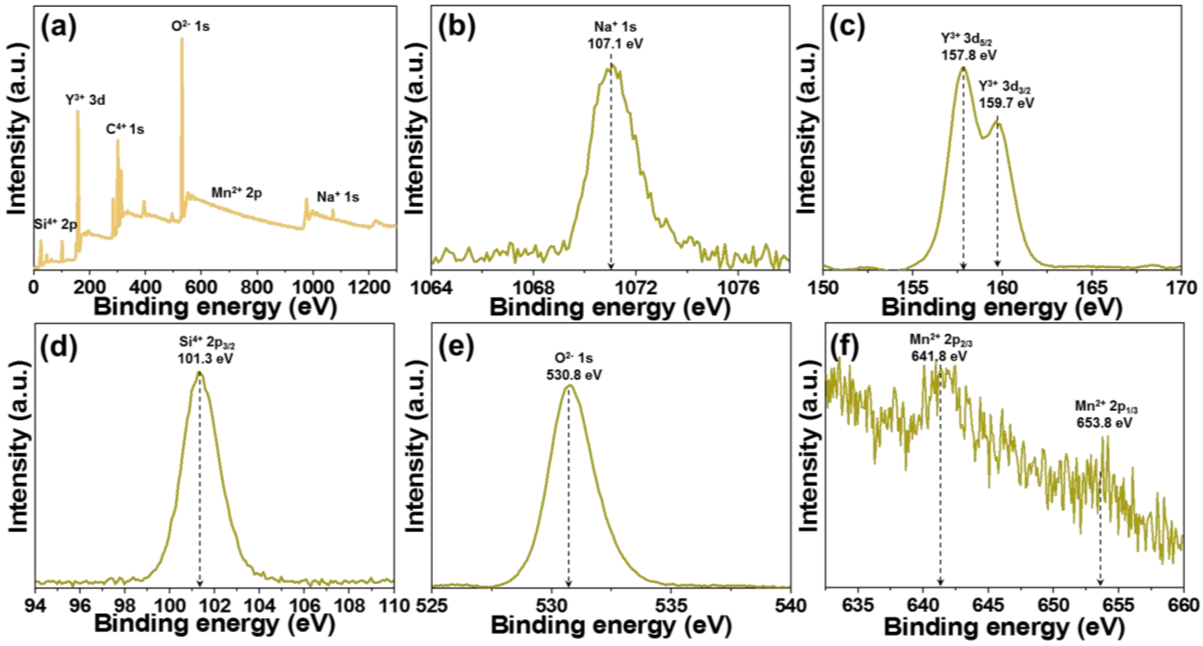

Figure S1 (a) Full XPS survey spectrum of $NaY_9(SiO_4)_6O_2$:0.08Mn^{2+} phosphors. High-resolution XPS spectra of (b) Na^+ 1s, (c) Y^{3+} 3d, (d) Si^{4+} 2p, (e) O^{2-} 1s and (f) Mn^{2+} 2p of $NaY_9(SiO_4)_6O_2$:0.08Mn^{2+} phosphors.

For the sake of obtaining the elemental information of studied samples, the typical XPS spectra of $NaY_9(SiO_4)_6O_2$:0.08Mn^{2+} phosphor were measured and shown in Figure S1. From the full XPS survey spectrum (Figure S1(a)), one knows that the elements of Na^+, Y^{3+}, Si^{4+}, O^{2-} and Mn^{2+} exist in the studied samples. It is shown in Figure S1(b) that the high-resolution XPS spectrum of Na^+ 1s is dominated by an intense band with the binding energy of 107.1 eV.[S3] The high-resolution XPS spectrum of Y^{3+} 3d consists of two bands at about 157.8 and 159.7 eV corresponding to Y^{3+} $3d_{5/2}$ and Y^{3+} $3d_{3/2}$, respectively, as shown in Figure S1(c).[S4] The high-resolution XPS spectrum of Si^{4+} 2P is prevailed by a strong band with the binding energy of 101.3 eV, which is assigned to the Si^{4+} $2P_{3/2}$ (see Figure S1(d)).[S5] Moreover, the band with the binding energy of 530.8 eV is attributed to the O^{2-} 1s (Figure S1(e)).[S6] As for the high-resolution XPS spectrum of Mn^{2+} 2P (see Figure S1(f)), it is made up two bands with the binding energies of 641.8 and 653.8 eV pertaining to the Mn^{2+} $2P_{2/3}$ and Mn^{2+} $2P_{1/3}$, respectively.[S7]

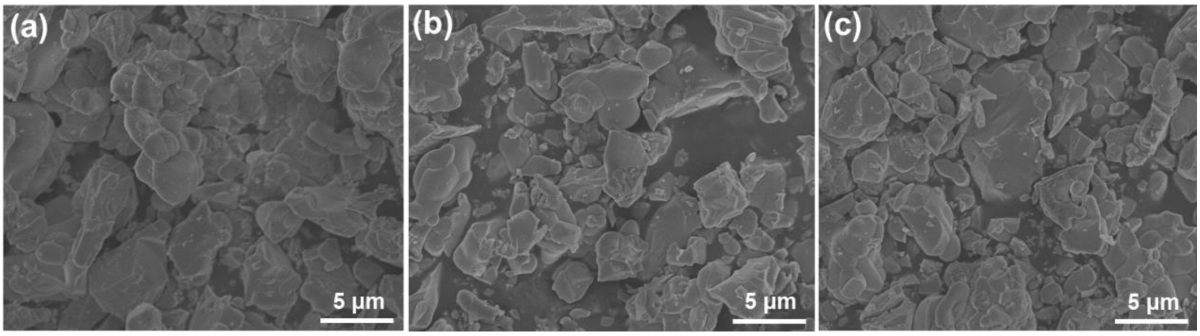

Figure S2. FE-SEM images of $NaY_9(SiO_4)_6O_2$:$x$$Mn^{2+}$ phosphors with Mn^{2+} content of (a) x = 0.02, (b) x = 0.04 and (c) x = 0.06.

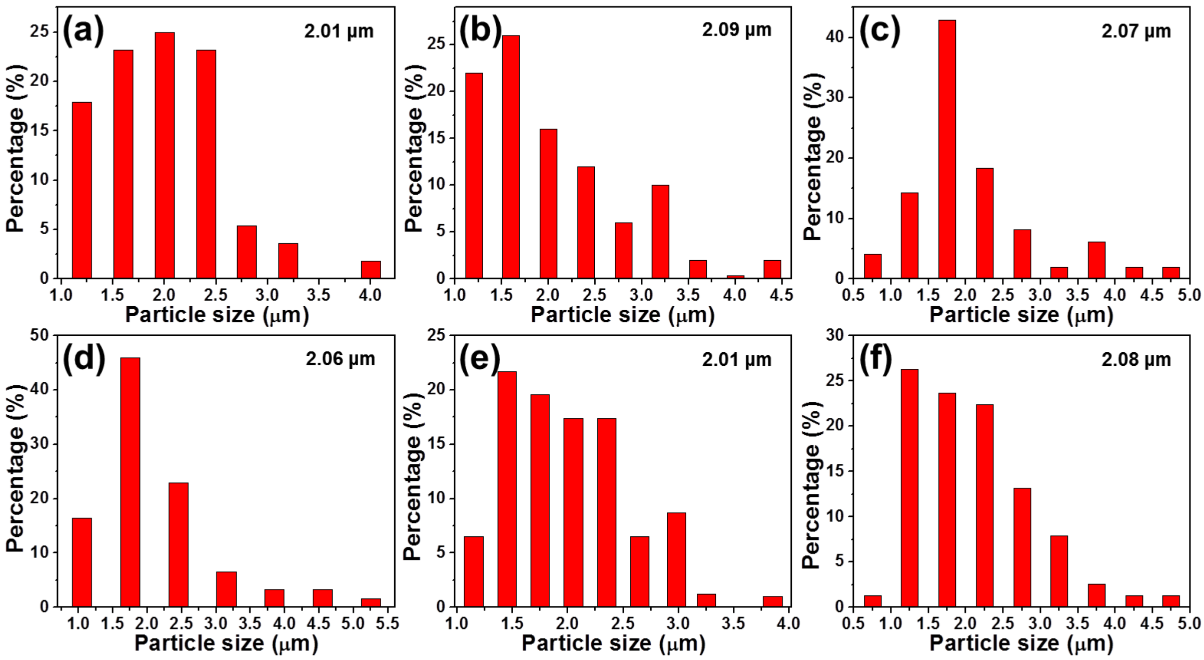

Figure S3. Particle distribution of $NaY_9(SiO_4)_6O_2$:$x$$Mn^{2+}$ phosphors doped with different Mn^{2+} contents of (a) x = 0.005, (b) x = 0.02, (c) x = 0.04, (d) x = 0.06, (e) x = 0.08 and (f) x = 0.10.

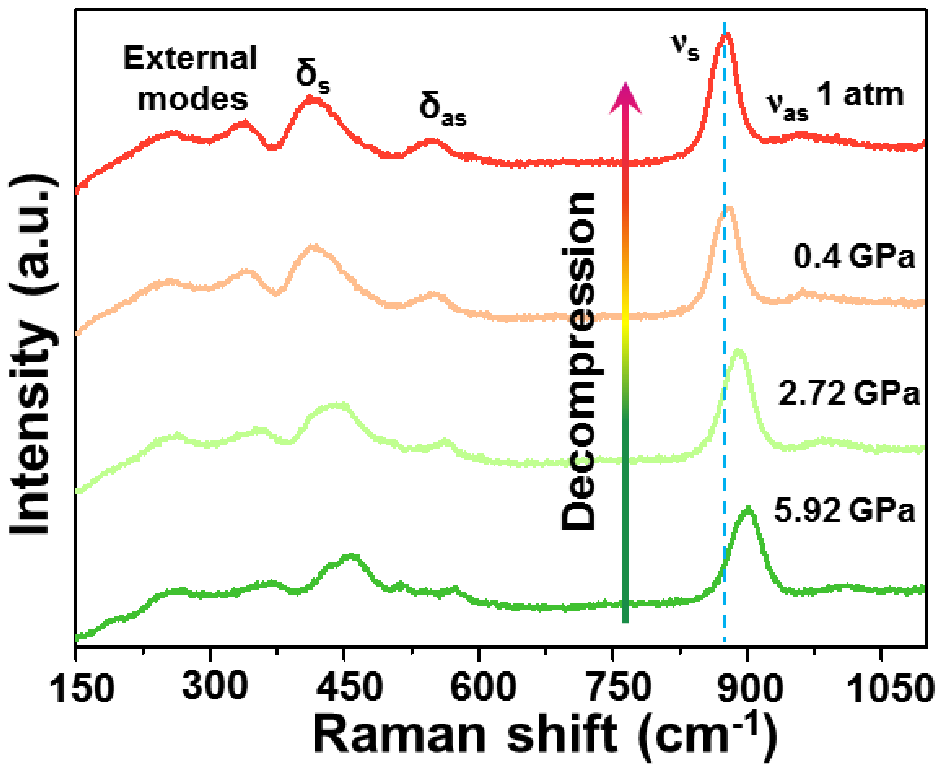

Figure S4. Pressure dependent Raman spectra of $NaY_9(SiO_4)_6O_2$:0.08Mn^{2+} phosphors during the decompression process.

References

[S1] J. Zhu, P. Du, J. Wang, *J. Lumin*. **2022**, *248*, 118929.

[S2] P. Du, Y. Hou, W. Li. L. Luo, *Dalton Trans*. **2020**, *49*, 10224-10231.

[S3] J. Tang, P. Du, W. Li, L. Luo, *J. Lumin*. **2020**, *224*, 117296.

[S4] S. Zhong, X. Wang, Y. Wang, F. Zhou, J. Li, S. Liang, C. Li, *J. Alloys Compd*. **2020**, *843*, 155598.

[S5] X. Li, X. Wang, R. Hu, Y. Li, X. Yao, *J. Alloys Compd*. **2022**, *896*, 162877.

[S6] W. Ji, M. Lee, L. Hao, X. Xu, S. Agathopoulos, D. Zheng, C. Fang, *Inorg. Chem*. **2015**, *54*, 1556-1562.

[S7] H. Dai, S. Li, Z. Li, J. Li, S. Xin, C. Wang, G. Zhu, B. Dong, *J. Am. Ceram. Soc*. **2022**, *105*, 4719-4730.
